# Identification of biofilm proteins in non-typeable *Haemophilus Influenzae*

**DOI:** 10.1186/1471-2180-6-65

**Published:** 2006-07-19

**Authors:** Timothy K Gallaher, Siva Wu, Paul Webster, Rodrigo Aguilera

**Affiliations:** 1 Proteomic Core Facility, School of Pharmacy, Health Sciences Campus, University of Southern California, Los Angeles, CA 90033, USA; 2 Ahmanson Advanced Electron Microscopy and Imaging Center, House Ear Institute, 2100 West Third Street, Los Angeles, CA 90057, USA

## Abstract

**Background:**

Non-typeable *Haemophilus influenzae *biofilm formation is implicated in a number of chronic infections including otitis media, sinusitis and bronchitis. Biofilm structure includes cells and secreted extracellular matrix that is "slimy" and believed to contribute to the antibiotic resistant properties of biofilm bacteria. Components of biofilm extracellular matrix are largely unknown. In order to identify such biofilm proteins an *ex-vivo *biofilm of a non-typeable *Haemophilus influenzae *isolate, originally from an otitis media patent, was produced by on-filter growth. Extracellular matrix fraction was subjected to proteomic analysis via LC-MS/MS to identify proteins.

**Results:**

265 proteins were identified in the extracellular matrix sample. The identified proteins were analyzed for COG grouping and predicted cellular location via the TMHMM and SignalP predictive algorithms. The most over-represented COG groups identified compared to their frequency in the *Haemophilus influenzae *genome were cell motility and secretion (group N) followed by ribosomal proteins of group J. A number of hypothetical or un-characterized proteins were observed, as well as proteins previously implicated in biofilm function.

**Conclusion:**

This study represents an initial approach to identifying and cataloguing numerous proteins associated with biofilm structure. The approach can be applied to biofilms of other bacteria to look for commonalities of expression and obtained information on biofilm protein expression can be used in multidisciplinary approaches to further understand biofilm structure and function.

## Background

Bacteria exist in both planktonic and biofilm states [1,2]. Recent findings indicate chronic infections are associated with the formation of *in vivo *biofilm which renders the bacteria resistant to antibiotic treatment [3]. This resistance has been believed to be due to the structural properties of the biofilm which have been described as "matrix encased microbrial communities" [4]. More recently, studies of *Pseudomonas aeruginosa *biofilm indicated that simple lack of anti-biotic penetration is not the cause of resistance [5] and "anoxic regions where bacteria are poorly killed due to very low metabolic rates" in has been hypothesized [6]. Formation of biofilm includes adherence events wherein the bacteria become sessile and secrete extracellular matrix. The end result is a highly structured multicellular complex with cavities and channels [2]. Historically, molecular and biochemical studies of bacteria have examined the planktonic state rather than biofilm state. Understanding the molecular nature of the biofilm structure is of interest in developing strategies to combat chronic biofilm infections.

## Results and discussion

**Figure 1:**
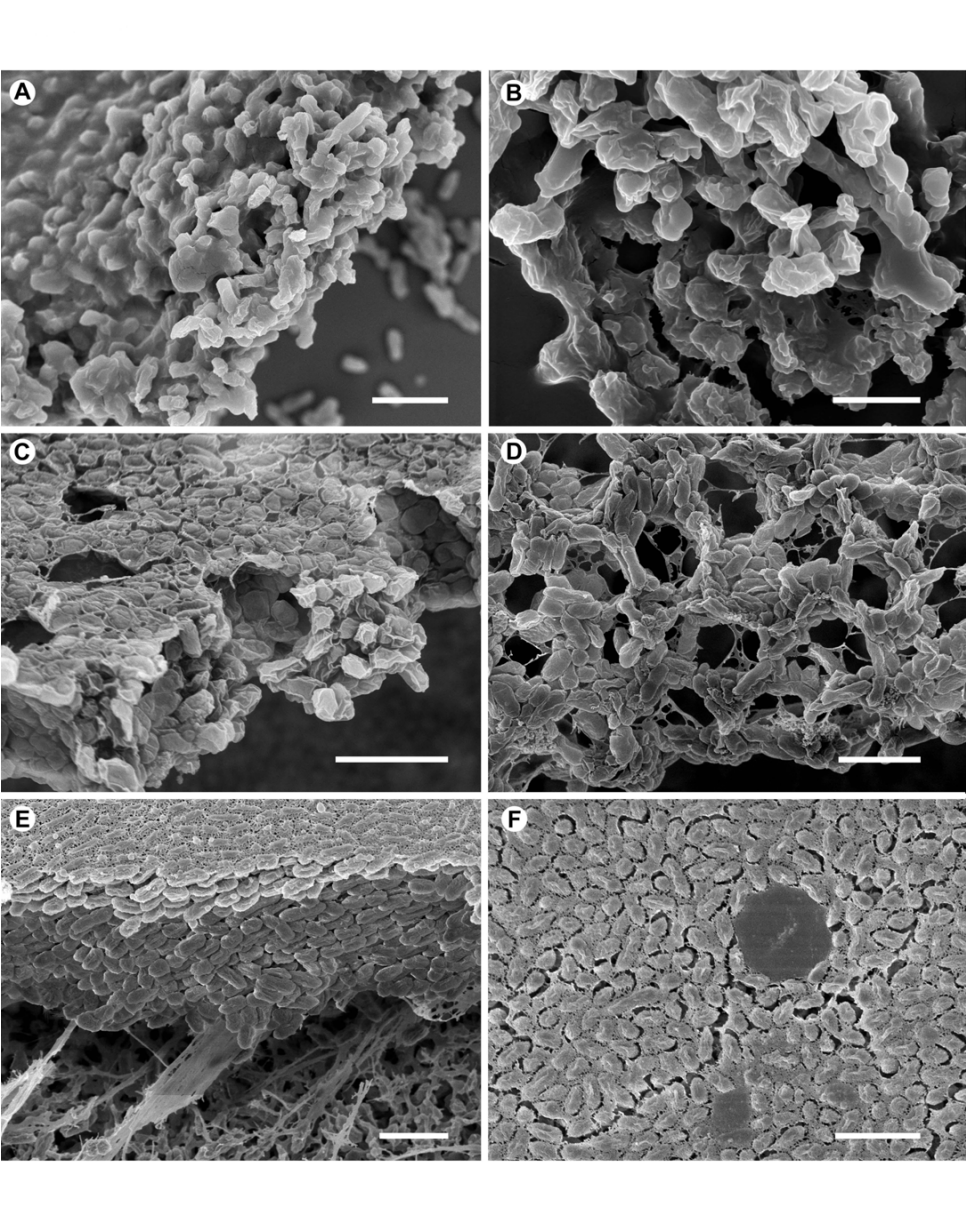
**Nontypeable *Haemophilus influenzae *biofilm imaged via scanning electron microscopy**. Scanning electron micrographs of NTHi biofilms formed under different growth conditions. A and B) Sterile glass coverslips were covered with a suspension of NTHi in BHI broth. After 24 hr, the coverslips were prepared for SEM examination. (A) Large flat mats of bacteria embedded in an amorphous extracellular matrix were found attached to the glass surface. Scale bar = 2 μm. (B) The individual NTHi are covered in an amorphous layer that conceals the bacterial surface. Scale bar = 1 μm. C and D) Suspensions of NTHi in BHI broth were placed onto sterile Anopore insert filters that were mounted on chocolate agar. Once the NTHi biofilms had formed, after 24 hr incubation, on the upper surface of the filters at the air/liquid interface, the inserts were placed in culture dishes containing sufficient sterile culture medium to exert a positive upward pressure on the bottom of the biofilm, and left for a subsequent 24 hr. (C) The surface of the insert filter is covered with a flat mat consisting of NTHi closely attached to each other. Channels and pockets freee of bacteria have formed within the mat of bacteria. Scale bar = 2 μm. (D) In some orientations, it is possible to see the channels running between the aggregates of bacteria and through the mat. Scale bar = 2 μm. E and F) NTHi biofilms grown on Millipore filters. Sterile Millipore filters were placed onto chocolate agar plates and inoculated with sufficient NTHi in BHI broth to cover the surface at a density of 0.3 bacteria per 10 μmP^2P^. The filters were incubated for 24 hr with the upper surface exposed to air, and prepared for SEM examination. (E) The NTHi formed thick biofilms with the base firmly attached to the filter substrate. Scale bar = 2 μm. (F) The top surface of the NTHi biofilm, that had been exposed to air, was covered with a thin film of extracellular matrix. In some instances, the matrix formed a film over regions that resembled bacteria-free pockets. Scale bar = 2 μm.

Non-typeable *Haemophilus influenzae*P (NTHi) is a gram-negative gamma-proteobacterium [7] that is the cause of otitis media (OM), a common chronic inner ear infection, and also sinusitis, bronchitis and other diseases, first demonstrated in a 1998 report [8] (see also a later review [9]). NTHi forms biofilm *in vitro *and NTHi isolates from children with otitis media and adults with chronic obstructive pulmonary disease have been shown to form biofilm in model systems [10] or *ex vivo *[11]. To address the question of biofilm extracellular matrix molecular structure, NTHi strain 9274, originally derived from an OM patient [12], was used to develop an *ex vivo *biofilm model wherein NTHi colony biofilm was formed on filter substrates placed on the surface of chocolate agar plates. Biofilm formation in this system has been extensively characterized previously [13]. Biofilm formed on glass, anopore filter and Millipore filter are shown in electron micrographs at differing magnifications (fig 1a–f) which illustrate the extensive structure formed by the NTHi bacteria. Visible in the EM's is the extracellular mucopolysaccharide layer that forms around bacteria in biofilm. This layer is observed to express lipooligosaccharide LOS (unpublished observations), consistent with, and seen before in, NTHi biofilm [11].

Extracellular matrix components were isolated by sonication and washing the biofilm growth filters, with the resultant wash centrifuged to remove any cellular debris or whole cell contamination. Electron micrographs of the protein sample supernatant and pellet are presented in supplemental material (supplemental figures 1 & 2 [see additional file 3]). The supernatant sample shows a fibrous appearance with no bacteria observed compared to the pellet fraction's granular appearance with visible bacteria. Protein constituents of the extracellular matrix were determined by LC-MS/MS analysis of SDS-PAGE resolved matrix component proteins. This proteomic approach was taken to identify as many proteins as possible present in the biofilm extracellular matrix. A simple methodology was utilized wherein SDS-PAGE gels were horizontally sliced into 20 sections. Each section was in-gel trypsin digested and subjected to LC-MS/MS analysis. MS/MS data were searched against a sequence file containing proteins of the four NTHi strain genome sequences available [7]. These strains are: KW20 [14], also known as Rd (lacks an important fimbrial gene cluster that is important for virulence as compared to type b strains *i.e*. capsule-lacking avirulent strain); R2846 (was isolated from middle ear fluid of a child with acute otitis media); R2866 (isolated from the blood of a child with meningitis) and 86-028NP [15] (a biofilm-forming clinical isolate from a pediatric patient with otitis media) [16]. 269 proteins from the *Haemophilus influenzae *biogroup aegyptius strain were also included in the search data file. Search against the full NCBI non-redundant protein database was also done. Identifications were made using Mascot as the primary search software [17].

**Figure 2:**
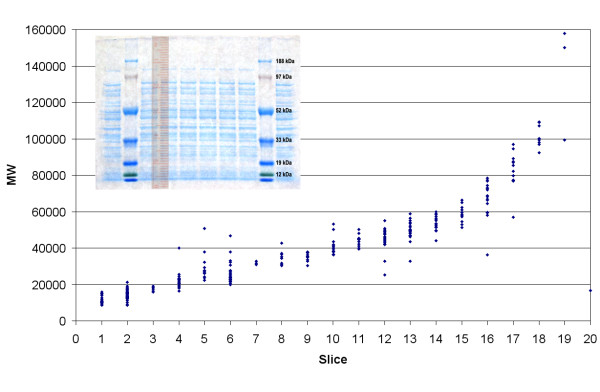
**Correlation of identified protein molecular weights with SDS-PAGE gel slices**. The x-axis indicates the molecular weight in Daltons of identified proteins in each gel slice. y represents the slice number of the gel from bottom of the gel (lower molecular weight) to the top of the gel (higher molecular weight).

For a protein to be identified and considered present, tryptic peptides corresponding to identified proteins had to be observed at or above the Mascot ions score cut-off and be the primary identification (or hit) with at least one peptide observed. In cases of single peptide identification, the MS/MS spectra were analyzed by the experimenters and all low scoring peptides were also analyzed in this manner. Representative chromatograms and MS/MS spectra are presented as supplemental figures 3 and 4, respectively [see additional file 3]. The conservative criteria for assigned identifications resulted in excellent correlation of molecular weight of the identified proteins with slice number (fig 2) which provides an indication of the integrity of the analysis. One exception was observed in that the protein identified in the topmost slice (slice 20), which should contain the highest molecular weight proteins in the sample, was an acyl carrier protein with a molecular weight near 17 kDa, much less than would be expected.

**Figure 3:**
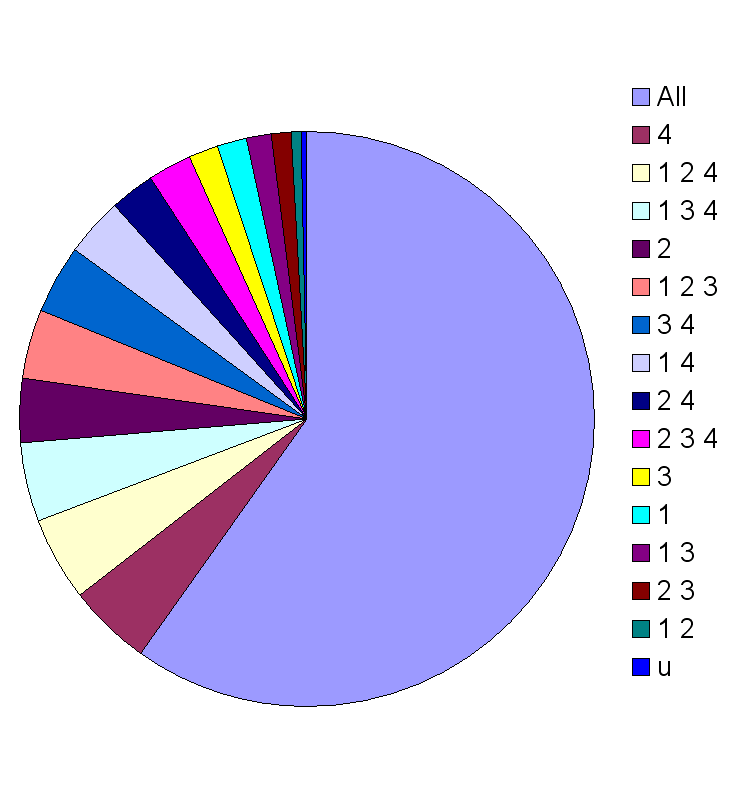
**Strain specific protein identifications in ECM biofilm sample**. All indicates no strain specific peptides were observed where the peptide sequence is present in homologous proteins of each of the four strains used for Mascot and Sequest searches. Strain key: 1: Rd aka KW20; 2: R2846; 3: R2866; 4: 86-028NP. U indicates one protein, a thioredoxin, with a peptide not found in any of the four strains searched. See reference 13 for strain information.

292 total protein identifications arising from analysis of all 20 gel slices were made with 27 being redundant (25 seen in two slices and one seen in three) for a total of 265 unique proteins identified in the extracellular matrix sample. Proteins corresponding to four of the five strains of NTHi proteins in the search data file were observed with the exception being the aegyptius strain. The analyte NTHi strain had been isolated from an OM patient [12] and is genetically uncharacterized. The majority of the proteins, 158, could not be assigned to a specific strain in that protein-identifying peptide sequences (or sequence tag) were shared by each of the four strains. Other identifying peptide sequences indicated that 32 strain-specific proteins were present with four proteins specific only for KW20, 10 specific for R2846, five specific for R2866 and 13 specific for 86-028NP. The other 77 identified proteins could be assigned to a combination of two or three strains. Determinants for strain specificity were usually based upon one amino acid difference in one of the protein-identifying peptides. The gene *ompA*, which codes for outer membrane protein P5, serves as an example. Strain KW20's OmpA contains glutamic acid at position 118 whereas each of the other strains has the conservative substitution aspartic acid at the corresponding position. The difference in mass of seven Da between the glutamic acid and aspartic acid-containing peptides in the doubly charged parent ion is easily resolved by MS and the corresponding CID fragmentation pattern subsequently obtained allows the specific identification. Figure 3 presents a distribution of the strain specificities observed for each protein. Tables containing all identified proteins are available as additional files in rtf format or tab-delimited text [stab2_rtf.rtf and stab2_txt.txt, respectively.]

A broader search using the full NCBI non-redundant database found only one non-HI peptide, a 14 amino acid peptide corresponding to a thioredoxin protein found in two Neisseria bacteria that differ from the corresponding HI thioredoxin peptide at three positions. This peptide and the mix of peptides corresponding to various annotated HI strain proteins most likely are representative of wild HI bacteria strain heterogeneity involved in chronic infection. Our observations of proteins corresponding to various HI strains, and in one case another species homolog, is supportive of and consistent with the idea of a supragenome "distributed throughout naturally occurring infectious populations" of HI, as hypothesized by Shen *et al *after a thorough sequence-based genetic analysis of 10 different clinical isolates of HI [18].

Observed in the biofilm ECM sample were a number of proteins annotated as uncharacterized, hypothetical or predicted coding region. 16 of these proteins were identified in our analysis and of these, ten of the 16 had previously been observed in two earlier NTHi proteomic analyses of the KW20 non-pathogenic strain in planktonic form [19,20], but six observed in this study are novel identifications via LC-MS/MS. Of these six, two had been identified in an even earlier proteomic study of planktonic KW20 which used 2-D electrophoresis combined with MALDI based protein fingerprinting and N-terminal sequence analysis [21]. Therefore four novel identifications of "hypothetical" NTHi proteins were obtained in this study. Annotation of clusters of orthologous groups (COG) [22], using COGnitor [23] if necessary, was done on all proteins (hypothetical/uncharacterized as well as named and described proteins). Of the 16 hypothetical/uncharacterized proteins, only six actually are in the COG category S (function unknown) and only one fell in to no COG category: the product of gene HI0246, a signal peptide-containing protein, returns no related COG using COGnitor. One other identified protein, the gene product of NTHI1707 (the 86-028NP strain homolog of HI1427) also is not assigned a COG category, although it is assigned COG5266 in its reference sequence entry, an ABC-type Co2+ transport system, periplasmic component. Table 1 presents information on these 17 proteins.

**Figure 4:**
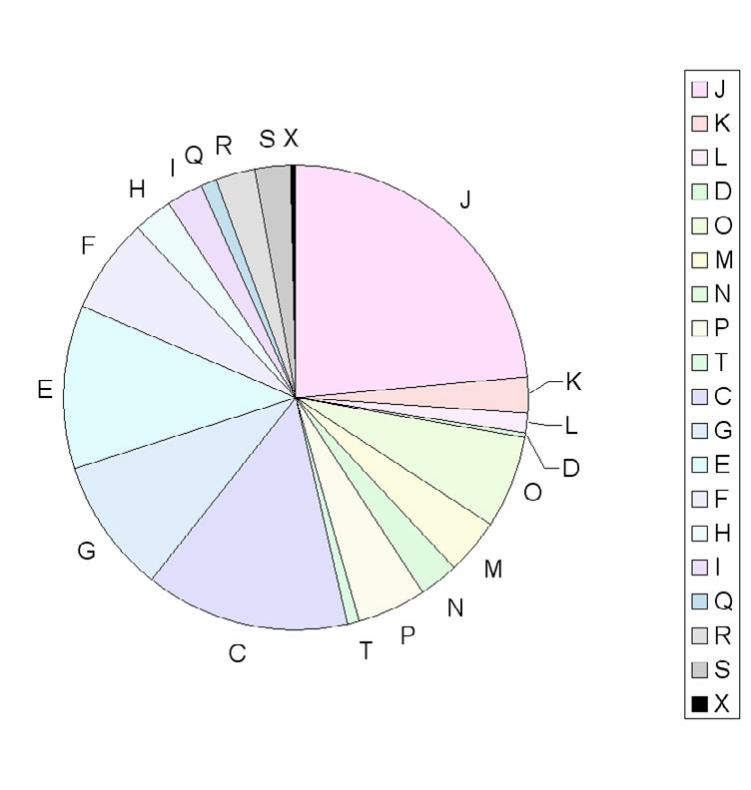
**COG Category distribution of identified biofilm ECM proteins**. The percent distribution of identified proteins in terms of their assigned COG categories. The chart is color-coded as per COG colors at the NCBI COG functional annotation siteP . COG category groupings are as follows: JKL – Information storage and processing; DOMNPT – Cellular processes; CGEFHIQ – Metabolism; RS – Poorly characterized. Categorization presented here reflects original COG categorization. In updated categorization P is included in Metabolism. X indicates a protein with no affiliated COG category.

**Figure 5:**
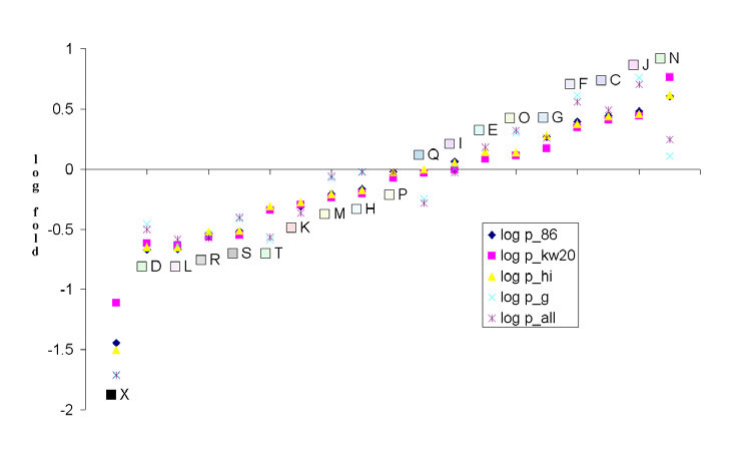
**Relative distributions of identified COG protein compared to genomic distributions**. Graphed from low to high are the % COG distribution in our identified sample compared to % COG distribution on bacterial genomes. The y-axis is the log of the ratio of COG % in our sample vs. COG % in a given genome. Diamond shows the distribution ratio in the 86-028NP genome; square is vs. Rd genome; triangle is all *Haemophilus influenzae *genomes; X is compared to all gammaproteobacteria genomes and the asterix is compared to all bacterial genomes. COG groups are labeled by number and color-coded as per the NCBI COG web page (as also in figure 4).

Full COG analysis showed that there was an overrepresentation of certain COG proteins compared to their genomic frequencies [24]. 67 ribosomal proteins (COG group J) were observed which accounts for 24% of the total proteins identified which is an overrepresentation compared to the genomic percentage of 7 – 8 % in HI. The most overrepresented group contained eight proteins identified (~3% of the total identified proteins) in COG group N (cell motility and secretion) but the HI genome comprises only ~0.7% of this COG group. Table 2 lists these COG group N proteins which includes an ABC type toluene transporter and the ClpP protein, which have been implicated in biofilm formation previously, [25,26] and also SecB, a chaperone upon which secretion of a number of proteins is dependent, including TolB which is also a member of COG group N and observed in this analysis [27]. In contrast the most underrepresented COG categories we observed were for COGs D and L, cell division and chromosome partitioning and DNA replication, recombination and repair, respectively. We observed only one protein for COG group D and four for COG group L. COG category distribution in our set of identified proteins by percent (fig 4) and relative to genomic distribution (fig 5) are presented.

All identifications were also screened for the presence of signal sequence secretory signal [28] and transmembrane domains [29]. 21 proteins were positive for signal sequence and 20 were positive for at least one TMH domain with six of these proteins containing both predicted signal sequence and TMD, often overlapping. These putative secreted or membrane bound proteins were in 12 different COG categories and also included the COG unclassifiable protein mentioned earlier. These proteins are included in supplemental table 1 [see additional file 3].

Given that the sample analyzed was preparatively isolated to be that which corresponds to extracellular matrix, we would have expected to have seen a larger proportion of secreted proteins, or possibly membrane proteins, in the analysis. We, though, identified large numbers of ribosomal proteins, metabolic enzymes or other proteins normally associated with intracellular localization and function. Electron microscopy of sample (presented in supplemental information; supplemental figures 1 and 2 [see additional file 3]) demonstrates that whole cell contamination of the sample has not occurred. The presence of lysed cell components cannot be ruled out. It is not known if such proteins act as components of the biofilm structure. The idea that these types of proteins may contribute to biofilm structure is possible in that dead cells and cell death have been reported to be part of biofilm structure and function [30,31] and an earlier proteomic approach in another bacteria, *Shewanella oneidensis *MR-1, identified ribosomal proteins as well in their analysis [32]. Further, proteins normally associated with intracellular function are observed outside the cell which has generated interest in a non-classical secretion pathway [33]. Among such normally intracellular proteins that have been demonstrated to also be found outside gram positive bacterial cells [33] are a number of proteins we have observed in this biofilm EMC sample, including ribosomal proteins, enolase, superoxide dismutase, elongation factor Tu and chaperonins DnaK and GroL (GroL is the COG gene name for GroEL proteins).

Overall 43 proteins (~16% of all identified proteins) are either annotated as periplasmic, membrane or membrane associated or were identified by signal peptide or TMH analysis (indicative of either periplasmic or membrane location). Nine annotated ABC transporters were identified in the sample which corresponds to 3.4% of the identified biofilm proteins. Eight additional proteins annotated transporters or periplasmic were also observed, including multi-membrane spanning transport proteins. Of note is that members of the ABC transporter protein class have been shown to be essential for biofilm formation including a membrane bound component of the ABC transporter in *Bacillus subtilis *[34], the lapEBC cluster of Pseudomonas fluorescens [35] and the *adc *operon of *Streptococcus gordonii *[36]. These 43 proteins are presented in supplemental table 1 [see additional file 3].

Recently, a chaperonin gene, *groEL1*, has been shown to be essential for biofilm formation in the gram-positive actinobacterium *Mycobacterium smegmatis *[37]. As mentioned, we see a groEl protein in our sample, and as noted above, GroEL is known to be localized outside bacterial cells as well as intracellularly. Our identification of groEL in biofilm sample ECM would seem to suggest a pan-bacterial role for groEl in biofilm formation, but there is a caveat. The groEL in HI is most similar to groEL2 of the mycobacterium, sharing a methionine-glycine rich carboxyl region whereas biofilm formation in the mycobacterium was attributed to the groEL1 gene, a homologous gene that has a histidine rich carboxyl terminus. Does the GroEL in HI or other bacteria which express only one GroEL form use this gene in biofilm formation? Of interest is that in this same study [37], GroEL2 is reported to physically associate with the protein KasA with the protein-complex levels being enhanced during biofilm formation. KasA is 3-oxoacyl-(acyl-carrier-protein) synthase, a FabB gene with a COG number 0304. In our sample the HI homolog is also present and is the earlier mentioned acyl carrier protein which migrated in SDS-PAGE at an anomalous molecular weight. A second protein reported to associate with KasA/FabB, referred to as SMEG4308 (COG0492), which has an HI homolog, was not seen in our analysis.

Also in our biofilm ECM sample is the universal stress protein UspA. A protein of the *uspA *family in *E. coli *has been shown to interact with and act as a substrate for GroEl-mediated phosphorylation [38]. Of further note is that, recently, UspA of the periodontopathic *Porphyromonas gingivalis *bacterium was reported to be necessary for biofilm formation [39].

Our analysis also identified two ompA outer membrane component proteins of NTHi, the P5 and P6 (also known as peptidoglycan-associated outer membrane lipoprotein). This class of outer membrane proteins can serve as adhesins and have been implicated in biofilm formation [11]. Of note in terms of pathogenicity and potential clinical issues is that P6 has been shown to induce human macrophage mediated immunogenicity associated with inflammatory events [40]. Recently ompA of *E. coli *has been demonstrated to regulate biofilm formation [41]. In this same study, two DNA binding transcriptional regulatory proteins, Hha and YbaJ of *E. coli *were also implicated in the biofilm formation. Hha positively regulates ompA expression. We did not see HHa or YbaJ in our analysis but did see two ompA proteins which is consistent with the ECM nature of our sample.

Another protein recently indicated to be found in NTHi biofilm cell envelope is a peroxiredoxin-glutaredoxin [42] which corresponds to HI0572 with COG number 678 and COG gene designation AHP1. The protein is observed to be present in greater abundance in biofilm and bacterial strains with expression deficient mutations showing a 25 – 50% decrease in biofilm formation. In our analysis this peroxiredoxin, AHP1, was identified, as was another peroxiredoxin, AHPC.

## Conclusion

This study has provided the results of an initial inquiry in to the protein structural components of biofilm. An *ex vivo *biofilm of NTHi bacteria (strain 9274), which was originally isolated from an otitis media patient, was grown on nitrocellulose membrane. Extracellular matrix proteins were isolated from the biofilm by sonication and washing of the filter and differential centrifugation and these proteins were resolved by SDS-PAGE and analyzed by LC-MS/MS ("proteomics"). In this manner 265 NTHi proteins were identified. Proteins identified indicated this isolate is a genetically unique strain (or non-clonal mix of strains) based upon sequences of identified peptides, sharing properties of four different well characterized (*i.e*. genomically sequenced) HI strains. All identifications are provided in supplemental information [see additional file 1 or 2].

Identified proteins were analyzed in terms of their COG group and functional categorization of COG, and ostensible cellular localization, *e.g*. presence of signal peptide or transmembrane helices or annotation indicating cellular localization. Hypothetical or uncharacterized proteins were characterized. Of these one was not able to be placed in a COG. Three were novel identifications for HI via the proteomic approach.

Importantly, a number of HI proteins homologous to proteins specifically implicated in biofilm formation in other bacteria were observed in our sample, including GroEL and a GroEl-associated acyl carrier, KasA/FabB, OmpA, UspA and peroxyredoxin.

This inquiry provides a starting point to further address questions of bacterial biofilm structure where information provided here can be applied in genetic, biochemical, biophysical or other types of studies. The method and information obtained also indicates how biofilms from other bacteria can also be evaluated and cross-correlated to answer broader questions of common biofilm structural components.

## Methods

### Biofilm and ECM protein isolation

NTHi biofilm growth and preparation has been characterized previously (13). Extracellular matrix proteins were isolated by briefly washing Millipore filter grown biofilm (fed on chocolate agar) in phosphate buffered saline (PBS) with sonication. This wash eluent was centrifuged to remove whole bacterial cells and supernatant was subjected to SDS-PAGE using Invitrogen NuPAGE 4 – 12%. Proteins were visualized by coomassie blue stain.

### LC-MS/MS: (a) In-gel tryptic digest

Protein bands from SDS-PAGE were excised from the gels and destained with 50% acetonitrile in 50 mM ammonium carbonate. In-gel tryptic digest was carried out using reductively methylated trypsin (Promega, Madison, WI). Prior to digestion, samples were reduced with DTT (10 mM in 50 mM ammonium carbonate for 60 minutes at 56°C) and subsequently alkylated with iodoacetamide (55 mM in 50 mM ammonium carbonate for 45 minutes in the dark at room temperature). The digestion reaction was carried out overnight at 37°C. Digestion products were extracted from the gel with a 5% formic acid/50% acetonitrile solution (2X) and one acetonitrile extraction followed by evaporation using an APD SpeedVac (ThermoSavant). The dried tryptic digest samples were cleaned with ZipTip (Millipore CB_18B_).

### (b) Analysis of tryptic peptides by tandem mass spectrometry for protein identifcation

The sample was resuspended in 10 μL of 60% acetic acid, injected via autosample (Surveyor, ThermoFinnigan) and subjected to reverse phase liquid chromatography using ThermoFinnigan Surveyor MS-Pump in conjunction with a BioBasic-18 100 × 0.18 mm reverse-phase capillary column (ThermoFinnigan, San Jose, CA). Mass analysis was done using a ThermoFinnigan LCQ Deca XP Plus ion trap mass spectrometer equipped with a nanospray ion source (ThermoFinnigan) employing a 4.5-cm long metal needle (Hamilton, 950-00954) in a data-dependent acquisition mode. Electrical contact and voltage application to the probe tip took place via the nanoprobe assembly. Spray voltage of the mass spectrometer was set to 2.9 kV and heated capillary temperature at 190 C. The column equilibrated for 5 min at 1.5 μL/min with 95% solution A and 5% solution B (A, 0.1% formic acid in water; B, 0.1% formic acid in acetonitrile) prior to sample injection. A linear gradient was initiated 5 min after sample injection ramping to 35% A and 65% B after 50 min and 20% A and 80% B after 60 min. Mass spectra were acquired in the m/z 400–1800 range.

### (c) Protein identification

Protein identification was carried out with the MS/MS search software Mascot 1.9 (Matrix Science) with confirmatory or complementary analyses with TurboSequest as implemented in the Bioworks Browser 3.2, build 41 (ThermoFinnegan).

## Abbreviations

Abbreviations are NTHi: Non-typeable *Haemophilus influenzae*; ECM: extracellular matrix; COG: Cluster of orthologous groups; LC-MS/MS: liquid chromatography tandem mass spectrometry; TMH/TMD: transmembrane helix or domain.

## Authors' contributions

PW and SW carried out the biofilm preparative and electron microscopy components of the study. TKG and RA carried out the proteomic aspects of the study. TKG did bioinformatic analysis and authored the paper. All authors read and approved the final manuscript.

## Figures and Tables

**Table 1:** Identified ECM biofilm proteins annotated as hypothetical or uncharacterized.

KW20 gene	gi	Strain	COG #	COG	Description	Gene	Prior ID	Prior ID
HI0121	1573076	all	773	M	UDP-N-acetylmuramate-alanine ligase	MurC	y	y
HI0145	1573101	All	3010	G	Putative N-acetylmannosamine-6-phosphate epimerase	NanE	N	N
HI0148	42628803	all	3055	S	Uncharacterized BCR	-	y	y
HI0203	1573163	All	80 6	J	RimM protein, required for 16S rRNA processing	RimM	N	N
HI0227	1573192	1 4	2731	S	Uncharacterized BCR	-	y	y
HI0246	1573213	1 3 4	0	x	No related COG	-	y	y
HI0719	1573721	all	251	J	Putative translation initiation inhibitor	TdcF	y	y
HI0760	1573769	all	2924	CO	Fe-S cluster protector protein	-	n	n
HI0847	1573861	all	3085	S	Uncharacterized BCR	-	n	y
HI1034	1574067	1 3 4	1666	S	Uncharacterized BCR	-	y	y
HI1168	68057915	all	2926	S	Uncharacterized BCR	-	y	y
HI1333	1574791	All	1534	J	Predicted RNA-binding protein containing KH domain, possibly ribosomal protein	-	N	N
HI1349	1574811	All	783	L	Starvation-inducible DNA-binding protein	Dps	Y	Y
HI1427	68058243	4	0	x	No related COG	-	y	y
HI1647	1574495	1 2 4	214	H	Pyridoxine biosynthesis enzyme	SNZ1	y	y
HI1681	53732528	2	3110	S	Uncharacterized BCR	-	y	y
HI1715	1574570	All	1949	F	Oligoribonuclease (3'->5' exoribonuclease)	Orn	N	Y

Proteins are listed in order of the gene number in the Rd strain genome (HI number). gi is the NCBI gene ID number; Strain is the specific strain as determined by peptide sequence (see figure 4 legend); COG# and COG cat were assigned by COGnitor; gene description is taken from COG number entry at NCBI; Prior ID cites whether or not the protein was identified in previous proteomic analyses of the Rd strain of HI with ID1 being a previous LC-MS/MS based study (22) and ID2 being a MALDI-TOF based study (23).

**Table 2:** Identified proteins of the most over-represented COG category.

gi	Protein	SigP	TMD	COG	COG Description	Gene
42629692	COG1862: Preprotein translocase subunit YajC [Haemophilus influenzae R2846]	n	y1	1862	Preprotein translocase subunit YajC	YajC
46129041	COG2854: ABC-type transport system involved in resistance to organic solvents, auxiliary component [Haemophilus influenzae R2846]	y	y1	2854	ABC-type exporter of toluene and related compounds, periplasmic component ABC-type exporter of toluene and related compounds, periplasmic component	Ttg2D
1573750	protein-export protein (secB) [Haemophilus influenzae Rd KW20]	n	n	1952	Preprotein translocase subunit SecB	SecB
46133774	COG0541: Signal recognition particle GTPase [Haemophilus influenzae R2866]	n	y1	0541	Signal recognition particle GTPase	Ffh
68057469	Outer membrane protein P4, NADP phosphatase [Haemophilus influenzae 86-028NP]	y	n	2503	Predicted secreted acid phosphatase	-
42630977	COG0740: Protease subunit of ATP-dependent Clp proteases [Haemophilus influenzae R2866]	n	n	0740	Protease subunit of ATP-dependent Clp proteases	ClpP
53733238	COG0823: Periplasmic component of the Tol biopolymer transport system [Haemophilus influenzae R2866]	n	y1	0823	Periplasmic component of the Tol biopolymer transport system	TolB
1573881	GTP-binding protein [Haemophilus influenzae Rd KW20]	n	n	1217	Predicted membrane GTPase involved in stress response	TypA

N. gi is the NCBI gene ID number; Protein is from the gi entry at NCBI; SigP indicates presence of a predicted signal sequence as determined by SignalP (28); TMD represents the presence of transmembrane domains as predicted by TMHMM (29); COG is the COG number for the identified protein with associated description and gene.

## References

[B1] Costerton JW, Stewart PS, Greenberg EP (1999). Bacterial biofilms: a common cause of persistent infections. Science.

[B2] Stoodley P, Sauer K, Davies DG, Costerton JW (2002). Biofilms as complex differentiated communities. Annu Rev Microbiol.

[B3] Fux CA, Costerton JW, Stewart PS, Stoodley P (2005). Survival strategies of infectious biofilms. Trends Microbiol.

[B4] Branda SS, Vik S, Friedman L, Kolter R (2005). Biofilms: the matrix revisited. Trends Microbiol.

[B5] Walters MC, Roe F, Bugnicourt A, Franklin MJ, Stewart PS (2003). Contributions of antibiotic penetration, oxygen limitation, and low metabolic activity to tolerance of Pseudomonas aeruginosa biofilms to ciprofloxacin and tobramycin. Antimicrob Agents Chemother.

[B6] Borriello G, Richards L, Ehrlich GD, Stewart PS (2006). Arginine or nitrate enhances antibiotic susceptibility of Pseudomonas aeruginosa in biofilms. Antimicrob Agents Chemother.

[B7] NCBI Taxonomy ID: 727. http://www.ncbi.nlm.nih.gov/Taxonomy/Browser/wwwtax.cgi?mode=Info&id=727&lvl=3&lin=f&keep=1&srchmode=1&unlock.

[B8] Rayner MG, Zhang Y, Gorry MC, Chen Y, Post JC, Ehrlich GD (1998). Evidence of bacterial metabolic activity in culture-negative otitis media with effusion. JAMA.

[B9] Murphy TF (2003). Respiratory infections caused by non-typeable *Haemophilus influenzae*. Curr Opin Infect Dis.

[B10] Ehrlich GD, Veeh R, Wang X, Costerton JW, Hayes JD, Hu FZ, Daigle BJ, Ehrlich MD, Post JC (2002). Mucosal biofilm formation on middle-ear mucosa in the chinchilla model of otitis media. JAMA.

[B11] Murphy TF, Kirkham C (2002). Biofilm formation by nontypeable *Haemophilus influenzae*: strain variability, outer membrane antigen expression and role of pili. BMC Microbiol.

[B12] Gu XX, Tsai CM, Apicella MA, Lim DJ (1995). Quantitation and biological properties of released and cell-bound lipooligosaccharides fromnontypeable *Haemophilus influenzae*. Infect Immun.

[B13] Webster P, Wu S, Webster S, Rich KA, McDonald K (2004). Ultrastructural preservation of biofilms formed by non-typeable *Hemophilus influenzae*. Biofilms.

[B14] Fleischmann RD, Adams MD, White O, Clayton RA, Kirkness EF, Kerlavage AR, Bult CJ, Tomb JF, Dougherty BA, Merrick JM (1995). Whole-genome random sequencing and assembly of *Haemophilus influenzae *Rd. Science.

[B15] Harrison A, Dyer DW, Gillaspy A, Ray WC, Mungur R, Carson MB, Zhong H, Gipson J, Gipson M, Johnson LS, Lewis L, Bakaletz LO, Munson RS (2005). Genomic sequence of an otitis media isolate of nontypeable *Haemophilus influenzae*: comparative study with *H. influenzae *serotype d, strain KW20. J Bacteriol.

[B16] Descriptions in parenthesis were taken from the Genome Project page for each of the strains. http://www.ncbi.nlm.nih.gov/entrez/query.fcgi?db=genomeprj&cmd=search&term=txid727[orgn].

[B17] Perkins DN, Pappin DJ, Creasy DM, Cottrell JS (1999). Probability-based protein identification by searching sequence databases using mass spectrometry data. Electrophoresis.

[B18] Shen K, Antalis P, Gladitz J, Sayeed S, Ahmed A, Yu S, Hayes J, Johnson S, Dice B, Dopico R, Keefe R, Janto B, Chong W, Goodwin J, Wadowsky RM, Erdos G, Post JC, Ehrlich GD, Hu FZ (2005). Identification, distribution, and expression of novel genes in 10 clinical isolates of nontypeable *Haemophilus influenzae*. Infect Immun.

[B19] Kolker E, Purvine S, Galperin MY, Stolyar S, Goodlett DR, Nesvizhskii AI, Keller A, Xie T, Eng JK, Yi E, Hood L, Picone AF, Cherny T, Tjaden BC, Siegel AF, Reilly TJ, Makarova KS, Palsson BO, Smith AL (2003). J Initial proteome analysis of model microorganism *Haemophilus influenzae *strain Rd KW20. Bacteriol.

[B20] Kolker E, Makarova KS, Shabalina S, Picone AF, Purvine S, Holzman T, Cherny T, Armbruster D, Munson RS, Kolesov G, Frishman D, Galperin MY (2004). Identification and functional analysis of 'hypothetical' genes expressed in *Haemophilus influenzae*. Nucleic Acids Res.

[B21] Langen H, Takacs B, Evers S, Berndt P, Lahm HW, Wipf B, Gray C, Fountoulakis  M (2000). Two-dimensional map of the proteome of *Haemophilus influenzae*. Electrophoresis.

[B22] Tatusov RL, Koonin EV, Lipman DJ (1997). A genomic perspective on protein families. Science.

[B23] Tatusov RL, Natale DA, Garkavtsev IV, Tatusova TA, Shankavaram UT, Rao BS, Kiryutin B, Galperin MY, Fedorova ND, Koonin EV (2001). The COG database: new developments in phylogenetic classification of proteins from complete genomes. Nucleic Acids Res.

[B24] COG genomic frequencies are available at the COG page of the organism's genome project. In this case strain 86-028NP was used. http://www.ncbi.nlm.nih.gov/sutils/coxik.cgi?gi=715.

[B25] Frees D, Chastanet A, Qazi S, Sorensen K, Hill P, Msadek T, Ingmer H (2004). Clp ATPases are required for stress tolerance, intracellular replication and biofilm formation in Staphylococcus aureus. Mol Microbiol.

[B26] O'Toole GA, Kolter R (1998). Initiation of biofilm formation in Pseudomonas fluorescens WCS365 proceeds via multiple, convergent signalling pathways: a genetic analysis. Mol Microbiol.

[B27] Baars L, Ytterberg AJ, Drew D, Wagner S, Thilo C, van Wijk KJ, de Gier JW (2006). Defining the role of the Escherichia coli chaperone SecB using comparative proteomics. J Biol Chem.

[B28] Bendtsen JD, Nielsen H, von Heijne G, Brunak S (2004). Improved prediction of signal peptides: SignalP 3.0. J Mol Biol Kolter.

[B29] Krogh A, Larsson B, von Heijne G, Sonnhammer ELL (2001). Predicting transmembrane protein topology with a hidden Markov model: Application to complete genomes. Journal of Molecular Biology.

[B30] Yarwood JM, Bartels DJ, Volper EM, Greenberg EP (2004). Quorum sensing in Staphylococcus aureus biofilms. J Bacteriol.

[B31] Webb JS, Thompson LS, James S, Charlton T, Tolker-Nielsen T, Koch B, Givskov M, Kjelleberg S (2003). Cell death in Pseudomonas aeruginosa biofilm development. J Bacteriol.

[B32] De Vriendt K, Theunissen S, Carpentier W, De Smet L, Devreese B, Van Beeumen J (2005). Proteomics of Shewanella oneidensis MR-1 biofilm reveals differentially expressed proteins, including AggA and RibB. Proteomics.

[B33] Bendtsen JD, Kiemer L, Fausboll A, Brunak S (2005). Non-classical protein secretion in bacteria. BMC Microbiol.

[B34] Branda SS, Gonzalez-Pastor JE, Dervyn E, Ehrlich SD, Losick R, Kolter R (2004). Genes involved in formation of structured multicellular communities by Bacillus subtilis. J Bacteriol.

[B35] Hinsa SM, Espinosa-Urgel M, Ramos JL, O'Toole GA (2003). Transition from reversible to irreversible attachment during biofilm formation by Pseudomonas fluorescens WCS365 requires an ABC transporter and a large secreted protein. Mol Microbiol.

[B36] Loo CY, Mitrakul K, Voss IB, Hughes CV, Ganeshkumar N (2003). Involvement of the adc operon and manganese homeostasis in Streptococcus gordonii biofilm formation. J Bacteriol.

[B37] Ojha A, Anand M, Bhatt A, Kremer L, Jacobs WR, Hatfull GF (2005). GroEL1: a dedicated chaperone involved in mycolic acid biosynthesis during biofilm formation in mycobacteria. Cell.

[B38] Bochkareva ES, Girshovich AS, Bibi E (2002). Identification and characterization of the Escherichia coli stress protein UP12, a putative in vivo substrate of GroEL. Eur J Biochem.

[B39] Kuramitsu HK, Chen W, Ikegami A (2005). Biofilm formation by the periodontopathic bacteria Treponema denticola and Porphyromonas gingivalis. J Periodontol.

[B40] Berenson CS, Murphy TF, Wrona CT, Sethi S (2005). Outer membrane protein P6 of nontypeable *Haemophilus influenzae *is a potent and selective inducer of human macrophage proinflammatory cytokines. Infect Immun.

[B41] Barrios AF, Zuo R, Ren D, Wood TK (2006). Hha, YbaJ, and OmpA regulate Escherichia coli K12 biofilm formation and conjugation plasmids abolish motility. Biotechnol Bioeng.

[B42] Murphy TF, Kirkham C, Sethi S, Lesse AJ (2005). Expression of a peroxiredoxin-glutaredoxin by *Haemophilus influenzae *in biofilms and during human respiratory tract infection. FEMS Immunol Med Microbiol.

